# Postpartum Cerebral Venous Thrombosis—A Single-Center Experience

**DOI:** 10.3390/brainsci11030327

**Published:** 2021-03-04

**Authors:** Zoltan Bajko, Anca Motataianu, Adina Stoian, Laura Barcutean, Sebastian Andone, Smaranda Maier, Iulia-Adela Drăghici, Rodica Balasa

**Affiliations:** 1 Department of Neurology, University of Medicine, Pharmacy, Science and Technology of Targu Mures, 540136 Targu Mures, Romania; zoltan.bajko@umfst.ro (Z.B.); anca.motataianu@gmail.com (A.M.); laura.barcutean@umfst.ro (L.B.); rodica.balasa@umfst.ro (R.B.); 2 Ist Neurology Clinic, Mures County Clinical Emergency Hospital, 540136 Targu Mures, Romania; adina.stoian@umfst.ro (A.S.); adn.sebastian007@gmail.com (S.A.); iuliab93@yahoo.com (I.-A.D.); 3 Department of Patophysiology, University of Medicine, Pharmacy, Science and Technology of Targu Mures, 540136 Targu Mures, Romania

**Keywords:** postpartum cerebral venous thrombosis, postpartum stroke, puerperium

## Abstract

Background: Cerebral venous thrombosis (CVT) is a rare variant of stroke in the general population, but an important subtype among pregnancy- and puerperium-related cases. Studies describing its risk factors and clinical characteristics are limited. The aim of our study is to disclose these aspects and compare with cases unrelated to pregnancy and puerperium. Materials and Methods: We performed a retrospective analysis including 88 consecutive cases from a tertiary neurology clinic with a diagnosis of CVT. Ten of the 88 cases (11.3%) appeared during the postpartum period. Results: The mean age of the puerperal CVT cases was 26.5 years. The main pregnancy-related risk factors besides puerperium were cesarean delivery (5/10), preeclampsia (2/10), and stillbirth (1/10). General risk factors for thrombosis, i.e., infection, smoking, and primary hypercoagulability, were identified in 50% of cases. Onset was in the first 3 weeks after delivery, with a mean value of 9.6 ± 5.6 days. Headache was present in 90% of postpartum CVT cases and in 76.1% of non-postpartum female cases. Seizures were more frequent in the postpartum group (60% vs. 34.8%). Onset was acute (<48 h) in 50% of postpartum cases and in 30.4% of the non-postpartum female group. The Rankin score at discharge was significantly lower in the postpartum group (0.22 vs. 0.7, *p* = 0.02), suggesting a more favorable short-term outcome. Conclusions: The early postpartum period represents an important risk for the development of CVT. Cesarean delivery and preeclampsia, besides general risk factors such as infection, smoking, and primary thrombophilia, contribute to enhanced risk. Puerperium-related CVT presents a more favorable outcome compared with CVT with other etiologies.

## 1. Introduction

Cerebrovascular disorders are uncommon but can have life-threatening and potentially disabling complications during pregnancy and the postpartum period. They pose significant risks for both the mother and child. They can be divided into two main categories: thrombosis and ischemia, whichinclude arterial and venous strokes and hemorrhage [1,2].

There is large variability in the incidence of stroke in pregnancy and puerperium, influenced by healthcare systems, geographical regions, study populations, and study designs. Based on a systematic review and meta-analyses, the pooled crude rate of stroke related to pregnancy was 30 cases per 100,000 pregnancies, with roughly equal rates between ischemic stroke, cerebral hemorrhage, and cerebral venous thrombosis (CVT) [3]. CVT accounts for 2–57% of strokes related to pregnancy [4,5,6,7,8,9,10], and the majority of cases appear in the postpartum period [11].

Pregnancy and postpartum period are associated with complex physiological changes and adaptation mechanisms, which can lead to altered homeostasis and a high risk of complications [1]. The main causes for increased CVT risk during the postpartum period are the hypercoagulability related to pregnancy, cesarean delivery, infections, blood loss during delivery and dehydration, fluctuations of intracranial pressure during labor, hypertensive complications of pregnancy, and even loss of cerebrospinal fluid after dural puncture [12,13]. The prothrombotic state during pregnancy results from a shift in the equilibrium between the fibrinolytic and hemostatic system to prevent severe hemorrhage during labor. There is an increased level of procoagulant factors, as well as a decreased level of anticoagulant factors such as protein S and C and reduced fibrinolytic activity. These hematological changes return to baseline only 6–8 weeks after delivery [14,15,16,17,18]. Acesarean section contributes to the higher rate of CVT in puerperium, probably because the surgically induced tissue destruction leads to increased thrombin generation, protein C activation, and accelerated clearance from plasma. The surgery-induced reduction of protein C levels is added to the pregnancy-related protein C resistance, resulting in increased risk of thrombosis [1].The pregnancy- and delivery-related anemia can lead to thrombocytosis, which is a risk factor for venous thrombosis [19].

Cerebral venous thrombosis is a rare cause of stroke in the general population affecting all age groups, but with important gender-related differences regarding the risk factor profile due to hormone-related risk factors. Because of the protean and nonspecific clinical presentation, the diagnosis is difficult, frequently delayed, and needs a high clinical awareness, but in case of early diagnosis and adequate treatment, the prognosis is much more favorable [20].

The aim of our study is to analyze the clinical characteristics, risk factors, and short-term prognosis of postpartum CVT.

## 2. Materials and Methods

### 2.1. Study Population

We performed a retrospective analysis including all patients from our neurology clinic with a diagnosis of CVT. We systematically searched our stroke database, focusing on June 2009 to December 2020.Our clinics consist of two parts: a general neurological department and a stroke unit. We provide treatment for two-thirds of the adult cerebral venous thrombosis cases in Mures County, Romania, with a population of 589,072 inhabitants, but also receive cases from the neighboring counties.

We included in the study all patients with CVT, except those with incomplete imagistic workup and questionable diagnoses.

The diagnosis of CVT was made on the basis of either magnetic resonance or computed tomography venous angiography, besides native and contrast-enhanced computed tomography (CT) or magnetic resonance imaging (MRI). We documented all available relevant clinical data of the selected patients focusing on pregnancy- and non-pregnancy-related risk factors, clinical characteristics, and prognosis. Severity of CVT was measured using the National Institutes of Health Stroke Scale (NIHSS) at admission and modified Rankin scale (mRS) at discharge.

The Institutional Review Board of our hospital approved our retrospective study (approval code: 31781).

### 2.2. Literature Search

For the literature review, we searched PubMed using the following keywords: “postpartum cerebral venous thrombosis” and “postpartum stroke.” We included only studies published in English with full-text versions available.

### 2.3. Statistical Analysis

For the patients’ baseline characteristics, we used descriptive statistic methods, expressed as means and standard deviations (SDs) or frequencies and percentages for continuous variables. The categorical variables were analyzed between the groups using a two-tailed Fisher’s exact probability test. Differences between groups were described using standard statistics, evaluated for significance using a two-sample *t*-tests or Mann–Whitney U test, depending on whether the distributional assumptions were satisfied judging by skewness–kurtosis tests.

## 3. Results

Among 13,000 stroke patients, we identified 93 cases with a diagnosis of CVT. Five patients were excluded because of incomplete imagistic workup. Of the 88 cases treated in the studied period, 63 were from our county. The incidence of CVT in our county was 1.39/100,000/year.

Ten (11.3%) of the 88 cases included in the study appeared during the postpartum period. None of the 88 cases presented with CVT during pregnancy.The proportion of women was 63.6% (56 cases); 21.4% (12) of them were on oral contraceptive medication.

### 3.1. Postpartum Group

Table 1 presents the main pregnancy-related and non-pregnancy-related risk factors and data related to pregnancy at onset after delivery and parity.

The mean age of the patients in the postpartum CVT group was 26.5 years, significantly lower compared with the non-postpartum female CVT group (39.8 years, *p* < 0.001). In 50% of the postpartum CVT cases, general risk factors for thrombosis were identified, i.e., infection, smoking, and primary hypercoagulability. In all cases, onset of CVT was in the first 3 weeks after delivery (early postpartum period) with a mean value of 9.6 ± 5.6 days.

### 3.2. Postpartum Versus Non-Postpartum Female Groups

Table 2 presents the main clinical and paraclinical characteristics of CVT in the postpartum and non-postpartum female patient groups.

Multiple cerebral veins were affected in more than 50% of cases in both the postpartum and non-postpartum CVT groups (60% and 52.1%, respectively). Superior sagittal sinus (SSS) was more frequently affected in the postpartum CVT group (60% vs. 34.7%) and the lateral sinuses (transverse and sigmoid sinuses) in the non-postpartum female group (76.1% vs. 50%). No deep cerebral venous, cavernous sinus, or cerebellar venous affection was recorded in the postpartum group. Headache was present at onset in 90% of postpartum CVT cases and in 76.1% of non-postpartum female cases. Seizures were more frequent in the postpartum group (60% vs. 34.8%), and hemiparesis occurred with similar frequency in both groups. Onset was acute in 50% of postpartum cases and in 30.4% of non-postpartum female cases, and subacute in 50% and 65.2%, accordingly, but the differences were not statistically significant

MRI venography as a diagnostic tool was more frequently used in the postpartum group (70% of cases) and CT venography in the non-postpartum group (58.7%). No catheter angiography was needed in the postpartum group and was performed in 6.5% of non-postpartum female cases.

The mean NIHSS was higher at admission in the non-postpartum group, but the difference was not statistically significant. The Rankin score at discharge was significantly lower in the postpartum group (0.22 vs. 0.7, *p* = 0.02), suggesting a more favorable short-term outcome.

There was no statistically significant difference regarding the D-dimer, cholesterol, and triglyceride levels, but hemoglobin and hematocrit were significantly lower and the thrombocyte count and erythrocyte sedimentation rate (ESR) were significantly higher in the postpartum group. D-dimer values were elevated in both groups.

### 3.3. Treatment

The mainstay of the treatment in both patient groups was parenteral anticoagulation with low-molecular-weight heparin (LMWH) in the acute phase followed by oral anticoagulation with vitamin K antagonist. Direct oral anticoagulants (DOACs) for long-term oral anticoagulation were used only in a minority of cases (one case in the postpartum group and twoin the non-postpartum female group used rivaroxaban). No endovascular interventions or decompressive surgery were performed.

The in-hospital mortality rate was zero in the postpartum group and 2.2% in the non-postpartum female group.

## 4. Discussion

The epidemiology of cerebral venous thrombosis has undergone significant changes over the last decade. According to recent publications, the incidence of the disease has increased in developed countries from 0.2–0.5 cases/100,000 inhabitants/year to 1.32–1.75/100,000/year [21,22,23,24]. The percentage of female patients also varies significantly between different countries, depending on the rate and type of oral contraceptives used. Earlier studies reported an almost three times higher incidence in women. Newer studies published from developed countries, for example from Finland, Norway and Australia, observed no major sex differences in the incidence of CVT. These observations may be explained by the common use of low-estrogen contraceptive medications, which imply a lower thrombotic risk [23,24]. In Romania, the rate of use of oral contraceptives is lower than the European average, which is also reflected in the lower percentage of women with CVT, having as a risk factor the oral contraceptive drugs [25]. However, the percentage of female patients in our whole CVT cohort (63.6%) is situated between the averages in the high- and low-income countries [21,22,23,24].

In recent years, CVT mortality has also shown a declining trend. Our low in-hospital mortality rate (0% in the postpartum group and 2.2% in the non-postpartum female group) is consistent with the recent literature data from the high-income countries [22].

All CVT cases in our cohort appeared during the early postpartum period; none of them were diagnosed during pregnancy or late puerperium. These results are in line with the literature data. For the calculation of CVT risk in pregnancy and puerperium, Silvies et al. performed a case-control study using data from five academic hospitals. They stratified data for pregnancy and puerperium and early (0–6 weeks) versus late (7–12 weeks) postpartum period. They found no association between pregnancy and CVT. There was a 10-fold increase of risk during puerperium. The highest risk was during the first 6 weeks; these patients presented a 19-fold increased risk [26].

Lanska et al. investigated the potential risk factors for peripartum and postpartum stroke and CVT using a large database, including 1,408,015 sampled deliveries. They calculated a nationally representative risk of 11.6 cases of CVT per 100,000 deliveries. Cesarean delivery, hypertensive disorders of pregnancy, and infections other than respiratory tract infections (pneumonia, influenza) were the most important covariates strongly associated with CVT [11].

Preeclampsia represents an additional risk factor for CST, secondary to nephrotic syndrome and subsequent hypercoagulability. Ten percent of the 181 cases of CVT in pregnancy and puerperium reported by Carrol were preeclamptic/eclamptic [27].

The clinical picture of CVT in pregnancy and puerperium is not significantly different from that in the general population. The major clinical presentations include headache, seizures, and focal neurological signs such as hemiparesis, aphasia, and encephalopathy. These depend on the location and extent of cerebral lesions and the location and number of cerebral veins affected [1]. Because of the non-specific clinical signs, the diagnosis needs high clinical awareness. The postpartum woman presents several possible causes for headache besides CVT, such as hormonal and other complex physiological changes, use of vasoactive medication, psychological stress, insomnia, irregular meals, and neuraxial anesthesia for labor [28,29]. Therefore, the differential diagnosis is broad, and in suggested cases the neuroimagistical examinations must be performed promptly to establish an early diagnosis.

Kashkoush et al. [30] published a pooled, systematic review including 50 articles containing data from 66 CVT cases related to pregnancy and puerperium. The mean age of the patients was 26.5 years, identical with our data. Thirty-six percent of patients were pregnant and 64 were in puerperium. Seventy percent of patients were primigravid. The most important clinical presentations were headache, in 73% of cases, seizures in 50%, motor weakness in 38%, coma or obnubilation in 45%, and visual problems in 24% of cases. The most frequently affected sinuses were SSS in 70% of cases and lateral sinuses in 58%. The deep venous system was affected in 13% of cases. In 56% of cases, multiple sinuses were affected. Associated thrombophilia was diagnosed in 38% of cases. Seven percent of patients presented with preeclampsia. Forty-five percent of patients underwent cesarean delivery, 26% were thrombolyzed, 8% were treated with endovascular intervention, and 8% underwent decompressive craniectomy. Good clinical outcome (mRS 0–2) was reached in 94% of cases. Mortality was 2% [30]. The majority of these observations are consistent with our finding, except for the absence of severe, comatose cases in our cohort requiring invasive interventions. The mortality rate in our cohort was 0%. These differences can be attributed to our smaller patient group.

The prognosis of pregnancy-related CVT is better compared with CVT cases in the general population, even if the severity of illness is similar in both populations. Cantu et al. published data from 67 patients with CVT associated with pregnancy and puerperium and compared these with 46 cases unrelated to pregnancy. The outcome was good in 80% of pregnancy-related cases and 58% of the non-pregnancy-related cases. The mortality was 9% and 33%, respectively [31]. More recent publications describe much better mortality rates [21,22,23,24].

Neuroimaging is the mainstay of the diagnosis and must be performed urgently in case of clinically suspected CVT. MRI and magnetic resonance (MR) venography are the first option, or CT and CT venography if the former is not available [32,33]. CT venography (CTV) allows the direct visualization of the filling defect suggestive of thrombosis. The accuracy of CTV is similar to time-of-flight (TOF) MR venography (MRV). It is more sensitive to superficial cerebral venous sinuses than in the case of deep venous affection [34,35]. The combination of native CT and CTV results in high accuracy of CVT diagnosis [36]. Of the MRV techniques, TOF is the most frequently used, can demonstrate absence of flow in cerebral venous sinuses, and is indispensable in CVT diagnosis in pregnant and breastfeeding women and in chronic renal failure, where contrast administration must be avoided. Contrast-enhanced MR venography provides higher sensitivity, because it can eliminate complex flow artifacts in TOF [35]. Nowadays, conventional angiography is used only in select cases, where noninvasive techniques offer inconclusive results, for example in the case of isolated cortical vein thrombosis [32].

The basis of CVT treatment is parenteral anticoagulation with unfractionated heparin (UFH) or low-molecular-weight heparin, followed by oral anticoagulation for 3–12 months. The European Stroke Organization guideline published in 2017 prefers LMWH instead of UFH because of the more predictable pharmacokinetics and better outcome [33,37]. For long-term anticoagulation, guidelines recommend only vitamin K antagonist or LMWH, because there were no randomized trials finalized with NOACS before. The novel, non-vitamin K antagonists are used in select cases off label for CVT indication; however, there is a randomized trial published in 2019 that proved the efficacy and safety of dabigatran for this indication [38].There are limited, but promising, data on factor Xa inhibitors from case studies and small case series [39,40,41,42]. Randomized trials are ongoing for the indication of CVT [43]. In pregnant and puerperal, breastfeeding women, the best option is LMWH treatment [44]; however, the current clinical evidence suggests that warfarin is not excreted into breast milk, and may be given safely to the postpartum women requiring anticoagulation [45].

The main limitations of our study are the retrospective character and the relative small cohort, arising from the single center design.

## 5. Conclusions

The early postpartum period poses an important risk for the development of cerebral venous thrombosis. Cesarean delivery and preeclampsia, besides general risk factors such as infection, smoking, and primary thrombophilia, contribute to enhanced risk in this setting. Because symptoms are nonspecific, it is important to sustain a high level of clinical suspicion when a puerperal woman presents with headache, seizures, focal neurological signs, and altered mental status. Puerperium-related CTV presents a more acute onset and more favorable outcome compared with CVT with other etiologies.

## Figures and Tables

**Table 1:** Postpartum patient group. The main pregnancy-related and general risk factors and data related to pregnancy as onset after delivery and parity.

Case Nr	Pregnancy-Related Risk Factors	General Risk Factors	Age	Onset after Delivery (Days)	Parity
1	puerperium cesarean delivery	-	21	9	3
2	puerperium cesarean delivery preeclampsia	-	25	5	1
3	puerperium	-	28	21	1
4	puerperium cesarean delivery	-	31	5	1
5	puerperium	-	25	12	1
6	puerperium cesarean delivery preeclampsia	-	28	10	1
7	puerperium cesarean delivery	infection	25	15	1
8	puerperium stillbirth at 29 weeks	smoking hyperhomocysteinemia APCR	33	7	3
9	puerperium	smoking	23	11	1
10	puerperium	infection	26	1	2
Mean ± SD			26.5 ± 3.6	9.6 ± 5.6	1.5 ± 0.8

APCR: activated protein C resistance.

**Table 2:** The main clinical and paraclinical characteristics of CVT in the postpartum and non-postpartum female patient groups.

		Postpartum CVT Group (N = 10)	Non-Postpartum Female CVT Group (N = 46)	*p* Value
Affected sinuses	Multiple veins	6 (60%)	24 (52.1%)	0.78
	Single vein	4 (40%)	22 (47.8%)	1.0
	Superior sagittal sinus	6 (60%)	16 (34.7%)	0.36
	Lateral sinuses (transverse and/or sigmoid)	5 (50%)	35 (76.1%)	0.57
	Inferior sagittal, Straight sinus	2 (20%)	4 (8.7%)	0.32
	Deep veins	0	3 (6.5%)	1.0
	Cortical veins	0	3 (6.5%)	1.0
	Cerebellar veins	0	0	1.0
	Jugular veins	0	1 (2.2%)	1.0
	Cavernous sinus	0	1 (2.2%)	1.0
Clinical manifestations	Headache	9 (90%)	35 (76.1%)	0.8
	Seizures	6 (60%)	16 (34.8%)	0.36
	Hemiparesis	2 (20%)	10 (21.7%)	1.0
	Aphasia	0	3 (6.5%)	1.0
	Coma	0	2 (4.3%)	1.0
	Visual disturbances	1 (10%)	2 (4.3%)	0.46
	Encephalopathy/mental status disorder	1 (10%)	3 (6.5%)	0.56
	Oculomotor palsies	0 (%)	4 (8.7%)	1.0
The mode of onset	Acute (<48h)	5 (50%)	14 (30.4%)	0.5
	Subacute (>48 h to 30 days)	5 (50%)	30 (65.2%)	0.77
	Chronic (>30 days)	0	2 (4.3%)	1.0
Diagnostic venous imaging (beside the native CT and MRI)	MRI venography	7 (70%)	25 (54.3%)	0.77
	CT venography	3 (30)	27 (58.7%)	0.52
	Catheter angiography	0	3 (6.5%)	1.0
Parenchymal lesion on imaging	Hemorrhagic lesion	4(40%)	8 (17.4%)	0.25
	Non-hemorrhagic lesion (edema, infarction)	6 (60%)	21 (45.7%)	0.76
In-hospital mortality		0	1 (2.2%)	1.0
Age		26.5 ± 3.6	39.8 ± 15.3	*p* < 0.001
NIHSS at admission		1.8 ± 2.4	2.6 ± 4.7	NS
Rankin Scale at discharge		0.22 ± 0.4	0.7 ± 1.1	*p* = 0.02
D-dimer		3.74 ± 1.7	2.7 ± 1.9	NS

CVT: cerebral venous thrombosis, NIHSS: National Institutes of Health Stroke Scale.

## Data Availability

Available from the corresponding author on reasonable request.
